# Evaluating the Safety and Quality of Diagnostic Colonoscopies Performed by General Surgeons: A Retrospective Study

**DOI:** 10.7759/cureus.38955

**Published:** 2023-05-13

**Authors:** Muhammad Saulat Naeem, Ayesha Farooq, Zoya Sadiq, Irfan Saleem, Muhammad Usman Siddique, Aqeela Shirazi, Shahid Farooq, Muhammad Z Sarwar, Abrar Ashraf Ali

**Affiliations:** 1 Surgery, Mayo Hospital, Lahore, PAK; 2 Medicine, Naeem Hospital, Gujranwala, PAK

**Keywords:** surgeons versus gastroenterologists, colonoscopy bowel preparation, analgesia in colonoscopy, surgeons and colonoscopy, failed colonoscopy, colonoscopy quality indicators

## Abstract

Introduction

Colonoscopy, which is a challenging procedure and requires adequate time to master the skill, is the procedure of choice to visualize colonic mucosa to rule out many colonic pathologies. There is a dearth of published information from real clinical experiences regarding successful procedures and limitations.

The end point of colonoscopy is the visualization of the cecal pole by intubating the cecum. Many Europeans and English health organizations recommend that the procedure should have a completion rate of around or above 90%. Gut preparation is an important determinant for a successful procedure and obviates the need for further invasive and/or expensive procedures such as imaging.

The majority of colonoscopies are being performed by gastroenterologists (GI) throughout the world, and the role of a surgeon as an endoscopist is in debate. Before this study, neither a retrospective nor a prospective evaluation of the general surgeon's (GS) endoscopy's quality and safety had been evaluated in our institution.

Material and method

This retrospective observational study was carried out from 1 January 2022 to 31 August 2022 in the Department of Surgery at Mayo Hospital, Lahore, to evaluate colonoscopy completion rates, reason for failure, and complications in terms of bleeding and perforation. All patients undergoing lower gastrointestinal endoscopy (LGiE), both elective and emergency, were included. Patients under 15 years of age and patients known to be hepatitis B-positive or hepatitis C-positive were excluded from the study. All relevant data were entered into a data sheet. Qualitative variables such as gender, cecal intubation, adjusted cecal intubation, gut preparation, reasons for failed colonoscopy, analgesia use, and complications (bleeding and perforation) were calculated as frequency and percentage. Quantitative data such as age and pain score were reported as mean and standard deviation (SD). Details obtained were tabulated and analyzed via the Statistical Package for Social Sciences (SPSS) version 29.0 (IBM SPSS Statistics, Armonk, NY).

Results

A total of 57 patient data were collected; 35.1% (n=20) were female, and 64.9% (n=37) were males. The cecal intubation rate (CIR) was 49.1% (n=28), and the adjusted rate was 71.9%, excluding incompleteness due to mass obstructing lumen, 8.8% (n=5); planned left colonoscopy, 7% (n=4); sigmoidoscopy, 3.5% (n=2); distal stoma scope, 1.8% (n=1); and colonic stricture, 1.8% (n=1). The prevalent reason for failed colonoscopy was inadequate gut preparation (15.8% {n=9}). Other reasons include patient discomfort, 3.5% (n=2); looping of scope, 7% (n=4); and acute colonic angulation, 1.8% (n=1). No complications were recorded.

Conclusion

This study shows that colonoscopy can be done by general surgeons safely and effectively with adequate training. High rates of cecal intubation emerge during colonoscopies performed under deep sedation and by skilled colonoscopists. Adequate bowel preparatory regimen is compulsory for quality procedure.

## Introduction

Colonoscopy plays a vital role in the diagnosis and early management of many colonic pathologies including colorectal cancer [1]. Colonoscopy is technically challenging and demands talent that is tough to develop. Despite an increased focus on endoscopic performance measures, such as rates of cecal intubation, there is a dearth of published information from real clinical experiences.

Cecal intubation is the process of inserting the endoscope's tip deeply enough into the cecum to reach the appendiceal opening. Cecal intubation was deemed successful when the ileocecal valve (ICV) and appendiceal orifice (AO), two colonoscopic markers, were visible. The English National Health Service (NHS) Bowel Cancer Screening Programme (BCSP) and the European Society of Gastrointestinal Endoscopy (ESGE) both demand a cecal intubation rate (CIR) above 90% as a minimum benchmark. Depending on whether it is employed in "screening" or "symptomatic" groups of individuals, the US Multi-Society Task Force on Colorectal Cancer proposes different benchmarks (95% and 90%, respectively) [1].

The endoscope might not be able to reach the cecum due to the patient's discomfort during the process. Patients may receive conscious or sound sedation to avoid this scenario [2]. Sedation techniques vary greatly between countries and regions. Around 10% of colonoscopies in the United Kingdom are done without sedation, less than 1% are done with propofol, and the rest are done with mild sedation [3]. The intubation of the cecum increases sensitivity and lowers costs by obviating the need for imaging tests or a second colonoscopy to view the whole colon. To prevent colorectal cancer and lower mortality, a thorough mucosal examination is required when screening for colorectal carcinoma. The level of preparation impacts the scope of the inspection, the length of the process, and the option to stop or postpone the colonoscopy early [4,5].

The majority of colonoscopies are being performed by gastroenterologists (GI) throughout the world, and the role of a surgeon as endoscopist is in debate [6]. Before this study, neither a retrospective nor a prospective evaluation of the general surgeon's (GS) endoscopy's quality and safety had been evaluated in our institution. Safety in colonoscopy is defined as the successful completion of procedure and no intraoperative or postoperative complication encountered, and quality in colonoscopy is described as the completion of procedure with the visualization of appendicular orifice and ileocecal valve. This study compares general surgeons to colorectal surgeons and gastroenterologists to assess the safety and efficacy of diagnostic colonoscopies.

## Materials and methods

This descriptive study was conducted in the Department of Surgery at Mayo Hospital, Lahore, to analyze colonoscopy success rates, failure factors, and complications in terms of bleeding and perforation. All patients undergoing lower gastrointestinal endoscopy (LGiE) from 1 January 2022 to 31 August 2022, both elective and emergency, were included. Patients under 15 years of age and patients known to be hepatitis B-positive or hepatitis C-positive were excluded from the study. Data from hospital records were collected for all patients undergoing LGiE considering the inclusion and exclusion criteria. All patients were admitted via the outpatient department and/or emergency department of the hospital.

A standard gut preparation consisting of four sachets of Movicol mixed in 1 L of water was consumed orally over a 24-hour period prior to the procedure. Per rectal sodium biphosphate enema was administered 12 hours before the procedure for preparation. Patients were advised to consume clear liquids a day before the procedure and stay nil per oral on the day of the procedure. The endoscopists assessed the quality of the bowel preparation as (1) adequate, if there was no fecal material in the colon or only a tiny amount of thin fecal material that could be easily suctioned; (2) inadequate, if there was semisolid fecal material or a significant volume of solid fecal material discovered; and (3) unknown, if not documented in the chart.

An analgesia regimen consisting of 5 mg of nalbuphine along with metoclopramide and 2 mg of midazolam was administered for pain control and topped up by 1-2 mg as per the needs of patients. LGiE was performed by the same team of consultant surgeons.

A performa was prepared for the collection of data. All relevant data, i.e., age, gender, cecal intubation, adjusted cecal intubation, adequacy of gut preparation, reason for failed colonoscopy, analgesia use, pain score using a visual analog scale (VAS), and complications such as bleeding and perforation, were entered into a descriptive data sheet.

Details obtained were tabulated and analyzed via the Statistical Package for Social Sciences (SPSS) version 29.0 (IBM SPSS Statistics, Armonk, NY). The primary outcome was to evaluate the cecal intubation rate (CIR) and adjusted cecal intubation rate. Based on their respective endoscopist's observations of the ileocecal valve and appendiceal orifice, each one recorded the cecal intubation. In all cases, the rate of cecal intubation was calculated. The adjusted cecal intubation rate was calculated by excluding examinations aborted before reaching the cecum due to obstruction secondary to mass or stricture, planned sigmoidoscopy or left colonoscopy, and/or distal stoma scope. The endoscopist's record was retrieved when the cecum could not be reached, and the causes of the incomplete inspection were classified as looping of scope, inadequate colon preparation, patient's discomfort, or acute angulation.

Qualitative variables such as gender, cecal intubation, adjusted cecal intubation, adequacy of gut preparation, reasons for failed colonoscopy, analgesia use, and complications (bleeding and perforation) were calculated as frequency and percentages. Quantitative data such as age and pain score (VAS) were reported as mean and standard deviation (SD). Chi-square test was used to determine the level of significance. P-value of less than 0.05 will be considered significant.

## Results

A total of 57 LGiEs were performed during the eight months of this study. All the LGiEs were performed by the same consultant surgeon team and assisted by the trainees, and data were subjected to statistical analysis; 35.1% (n=20) were female, and 64.9% (n=37) were males. The mean age of the subjects was 40.9 years (SD, 16.5 years; range, 13-80 years); 66.7% (n=38) of patients had no previous surgery done, while 33.3% (n=19) of patients had abdominopelvic surgeries done prior to LGiE. The procedures done are shown in Table 1.

**Table 1:** Prior Surgical Procedures Undertaken in Patients Undergoing Colonoscopy

Surgical History	Frequency	Percentage
Colostomy reversal	2	3.5
Hemicolectomy with stoma	2	3.5
Laparoscopic appendectomy	1	1.8
Laparotomy with stoma	4	7.0
Left hemicolectomy	1	1.8
Left nephrectomy	1	1.8
No procedure	38	66.7
Rectal repair	2	3.5
Rectopexy	1	1.8
Rectovaginal fistula repair	2	3.5
Undocumented	3	5.3
Total	57	100.0

The most frequent reasons for a colonoscopy were rectal bleeding (21.1% {n=12}), followed by abdominal/pelvic mass (19.3% {n=11}) and abdominal pain (15.8% {n=9}). Other indications are shown in Table 2 along with their percentage of occurrence.

**Table 2:** Indications of Colonoscopy in Surgical Patients

Indications	Frequency	Percentage
Abdominal pain	9	15.8
Abdominal/pelvic mass	11	19.3
Constipation	6	10.5
Diarrhea	1	1.8
Diversion ileostomy	1	1.8
Enterocutaneous fistula	3	5.3
Follow-up case of hemicolectomy	2	3.5
Follow-up case of rectal perforation	2	3.5
Per rectal bleed	14	24.6
Perianal fistula	2	3.5
Rectal prolapse	4	7.0
Rectovaginal fistula	2	3.5
Total	57	100.0

The cecal intubation rate (CIR) was 49.1% (n=28), and the adjusted cecal intubation rate was 71.9% (excluding incompleteness due to mass obstructing lumen, 8.8% {n=5}; planned left colonoscopy, 7% {n=4}; sigmoidoscopy, 3.5% {n=2}; distal stoma scope, 1.8% {n=1}; and bowel stricture, 1.8% {n=1}).

Considering the adjusted cecal intubation rate, the major reasons encountered in this study for incomplete procedure were inadequate gut preparation (15.8% {n=9}) and looping of the scope (7% {n=4}). A detailed list of causes identified for incomplete procedure is shown in Table 3.

**Table 3:** Causes of Colonoscopy Failure With Estimated Frequency

Colonoscopy Failure Reasons	Frequency	Percentage
Distal stoma scope	1	1.8
Acute angulation	1	1.8
Inadequate gut preparation	9	15.8
Intubation done	28	49.1
Left colonoscopy	4	7.0
Looping of the scope	4	7.0
Mass obstructing lumen	5	8.8
Patient discomfort	2	3.5
Sigmoidoscopy	2	3.5
Stricture	1	1.8
Total	57	100.0

The CIR when compared with the patient's pain scores and discomfort showed that out of 28 patients who had cecal intubation done, 20 patients had mild pain, eight patients had moderate pain, and none of the patients had severe pain scores. While out of 29 patients in whom intubation was not achieved, nine patients had mild pain, 17 had moderate pain, and three had severe pain during the procedure, which was statically significant (P-value of 0.006). In comparing cecal intubation with the adequacy of gut preparation, the results showed that the proportion of cecal intubation achieved was more in patients who had adequate bowel preparation with statistically significant P-value of 0.04.

The major findings of the colonoscopy in surgical patients are patchy mucosal inflammation (17.5% {n=10}), rectal mass (12.3% {n=7}), and colonic stricture, fibrotic rent at rectosigmoid junction, hemorrhoids, rectal polyps, and solitary rectal ulcer (3.5% {n=2} each). The complete details of the findings are listed in Table 4.

**Table 4:** Colonoscopy Findings in Surgical Patients

Procedure Findings	Frequency	Percentage
Colonic stricture	2	3.5
Colonic worms	1	1.8
Enterocutaneous fistula	1	1.8
Fibrotic rent at rectosigmoid junction	2	3.5
Hemorrhoids	2	3.5
Incomplete procedure	10	17.5
Left colonic mass	2	3.5
Left colonic mass+enterocutaneous fistula	1	1.8
Patchy mucosal inflammation	10	17.5
Rectal mass	7	12.3
Rectal polyp	2	3.5
Rectal prolapse	1	1.8
Solitary rectal ulcer	2	3.5
Unremarkable	14	24.6
Total	57	100.0

A total of 50.9% (n=29) of patients had adequate gut preparation, and 42.1% (n=24) has inadequate gut preparation, while gut preparation adequacy was not documented for 7% (n=4); 71.9% (n=41) of patients did not receive any analgesia for the procedure, while 28.1% (n=16) of patients receive sedation and analgesia. Patient pain perception was assessed using a visual analog scale (VAS) pain score; the mean pain score was 3.74 (SD, 1.48; range, 2-9) and is tabulated in Table 5.

**Table 5:** Patient Pain Perception Assessed Using Visual Analog Scale Pain Score

VAS Pain Score	Frequency	Percentage
2	10	17.5
3	19	33.3
4	16	28.1
5	6	10.5
6	3	5.3
7	1	1.8
8	1	1.8
9	1	1.8
Total	57	100.0

Moreover, patients with adequate bowel preparation had less pain than those whose bowel preparation was inadequate with a significant P-value of 0.001, while the use of analgesia did not show any significance compared to no analgesia use with a P-value of 0.5 in achieving cecal intubation.

There were no complications recorded post procedure. Two patients had post-procedure abdominal distension and pain. Both had post-procedure abdominal and chest X-ray to rule out perforation, which came unremarkable. They had been given light meal after two hours and discharged home after six hours of observation.

## Discussion

Colonoscopy is the investigation and treatment modality for colorectal screening, anemia, cancer screening after polypectomy, cancer screening after resection, ulcerative colitis surveillance, and neoplastic masses. Typically, a colonoscopy allows for the inspection of the whole colon and the distal part of the terminal ileal mucosa. The intubation of the whole colon and mucosal visualization are essential components of a thorough evaluation of the large intestine [4,5]. Considering surgical setup, the common indications encountered for the procedure in our study were rectal bleeding, abdominopelvic mass, abdominal pain, constipation, rectal prolapse, and enterocutaneous, rectovaginal, and perianal fistula in decreasing frequency.

Ninety percent cecal intubation rate (CIR) is advised by international recommendations during a normal colonoscopy [7,8]. However, it has been noted in the literature that the CIR varies between 55% and 98.8% [4]. However, about 5%-10% of colonoscopies conducted by skilled endoscopists fail to intubate the ileum [2]. In the current study, the adjusted cecal intubation rate was 71.9%, which correlates with the CIR range described by Muslim and Al-Obaidi [4].

Even though skilled colonoscopists have reported high rates of cecal intubation, a tiny percentage of colonoscopies nevertheless end in failure. The CIR is influenced by a number of variables, including examiner-, patient-, and technique-related variables. Age, gender, body mass index (BMI), bowel habits, colonic diverticular disease, previous abdominal and pelvic surgery, and colon preparation quality are patient-related factors. The CIR is also influenced by the endoscopist's experience and volume of procedures. The use of sedative drugs and the choice of colonoscopy instrumentation are two aspects of the technique [9].

Excessive loop formation and failure to cross angulated, fixed, or strictured sigmoid are two causes of failure. Female patients who have had gynecological surgery in the past and with advanced diverticular disease are most likely to experience procedure failure [2,10]. Age and a greater body mass index (BMI) both enhance the likelihood of reaching the cecum. Young, healthy patients are more likely to have success with cecal intubation [1,11]. Poor bowel preparation is a considerable source of severe limitation on the effectiveness of colonoscopies. Adequate preparation helps in the good visualization of mucosa and results in greater ability to detect lesions [12-14]. We attribute the failure rate in the order of decreasing frequency to inadequate bowel preparation, mass obstructing lumen, excessive loop formation, patient's discomfort, and acute angulation.

According to Radaelli et al., the CIR in colonoscopies with and without sedation is, respectively, 84.2% and 76.1% [10]. This rate is quite low when compared to ours; the use of analgesia did not show any significance compared to no analgesia use with a P-value of 0.5 in achieving cecal intubation.

Using analgesic-assisted benzodiazepines during a colonoscopy is the most typical sedative strategy [15]. Propofol-based deep sedation was started to be utilized recently because it has been demonstrated that propofol has a better pharmacokinetic profile than benzodiazepines and opioids [16]. In light of the CIR and the absence of complications in any of the subjects, Tardu et al. concluded that propofol-based sedation is both secure and efficient [2]. The analgesia regimen used in our patients consists of 5 mg of nalbuphine along with metoclopramide, and 2 mg of midazolam was administered for pain control and topped up by 1-2 mg as per the needs of patients. Only 28.1% (n=16) of patients receive sedation and analgesia, and the mean pain score was 3.74 (SD: 1.48). Moreover, patients with adequate bowel preparation had less pain than those whose bowel preparation was inadequate with a significant P-value of 0.001, while the use of analgesia did not show any significance compared to no analgesia use with a P-value of 0.5.

Mehran et al. compared three groups, gastroenterologists (GI), colorectal rectal surgeons (CRS), and general surgeons (GS) performing colonoscopy, in their study and found no differences in the complication rate. However, GS tend to have a lower incomplete colonoscopy rate (0.32%) compared to CRS (0.84%) and GI (0.36%) (p=0.07). GS were faster with high completion rate and low morbidity rate. Therefore, they implied that general surgeons should compete for endoscopy suites [6].

According to Matyja et al., the rate of cecal intubation increased from 69.75% to 95.17% over the course of 14 years when using colonoscopes with responsive insertion technology (RIT), a novel combination of three technologies that includes passive bending, high-force transmission, and variable stiffness [1]. However, in this study, the cecal intubation rate was 49.1% (n=28), and the adjusted cecal intubation rate was 71.9%, which is lower than recommended.

Aslinia et al. [7] reported similar findings as per Matyja et al. [1] that the adjusted cecal intubation rate increased over the recent year. They discovered no association between the frequency of cecal intubation, patient age, the involvement of gastroenterology fellows, endoscopist's experience, or annual procedure volume. Contrarily, a higher probability of cecal intubation was predicted in colon cancer screening, male gender, outpatient colonoscopy, and sufficient bowel preparation [7].

According to Muslim et al., adjusted rates for cecal and ileal intubations were 94.2% and 50.8%, respectively. Overall, cecal and ileal intubation rates were reported to be 88% [4].

Cecal intubation and the avoidance of perforation are technical skills that improve with higher volumes and experience. On the other hand, low-volume colonoscopists require observation to minimize complications. Individual endoscopy centers might consider limiting their exposure to screening patients, when the consequences of perforation seem disastrous, and high risk colons. Low-volume colonoscopists might be encouraged to avoid struggling to accomplish cecal intubation and get help early with technically complex colons [17]. However, in our study, no complications were encountered.

The CIR increased with each additional 100 annual procedures (odds ratio {OR}: 1.17). Overall, adverse events trended lower with each 100 annual procedures (OR, 0.95; 95% confidence interval, 0.90-1.00). The risk of perforation appeared more clearly reduced, based on two large studies that had specifically examined this, with 8% and 4% risk reductions per 100 additional annual procedures [17].

The limitations of this study include its retrospective nature and the limited number of participants. As this study is based on a single-center observation, the results could not be generalize; hence, this study could act as a basis for multicenter analysis.

Medical knowledge is advancing quickly, and new methods are being developed daily. Gastroenterologists have a natural affinity for colonoscopy capabilities, and surgeons are quickly establishing sub-specialty interests. As a result, surgeons may worry about the safety and effectiveness of colonoscopies they perform given their lack of exposure. The introduction of natural orifice transluminal endoscopic surgery (NOTES) has however rekindled interest in endoscopy among surgeons. In order to improve their practice, we believe that the findings of our study will inspire our surgical colleagues to enhance these skills.

## Conclusions

Worldwide, LGiE are routinely performed by gastroenterologists. Many general and colorectal surgeons in the United Kingdom and Europe indulge in endoscopy practice. Surgeons in South Asia are showing a growing interest in performing endoscopic procedures for surgical patients. This study shows the safety and quality of endoscopic procedures performed by general surgeons in comparison with surgeons and gastroenterologists around the world.

Colonoscopy can be done by general surgeons safely and effectively with adequate training. High rates of cecal intubation emerge during colonoscopies performed under deep sedation and by skilled colonoscopists. Adequate bowel preparatory regimen is compulsory for quality procedure.
